# Anterior displacement of an avulsed posterio-latero-central tibial plateau fracture repaired with Herbert screws

**DOI:** 10.1016/j.amsu.2022.103928

**Published:** 2022-06-08

**Authors:** Abdullah AL sultan, Mohamad Al Masri, Mohammad Weam Bolad, Muhammad Al Atrash, Mohammad Alsultan

**Affiliations:** a Department of Orthopedic Surgery, Al Assad and Al Mouwasat University Hospitals, Damascus University- Faculty of Medicine, Damascus, Syria; b Department of Orthopedic Surgery, Damascus University- Faculty of Medicine, Khorfakkan Hospital, Sharjah, United Arab Emirates; c Department of Nephrology, Al Assad and Al Mouwasat University Hospitals, Damascus University- Faculty of Medicine, Damascus, Syria

**Keywords:** Posterio-latero-central tibial plateau fracture, Avulsion fracture, Free fragment, Anterior displacement, Herbert screws

## Abstract

**Introduction:**

Tibial plateau fractures are very complex articular fractures with a rare incidence of approximately 10.3%. These fractures may constitute a severe injury to the knee joint and other complications.

**Presentation of case:**

A 23- year-old male injured his right knee after a crush accident and was diagnosed with a posterio-latero-central (PLC) sheared fracture of the tibial plateau without an injury to knee ligaments. CT with a reconstruction of the right knee showed that the fracture was accompanied by a free fragment inserted into the anterior compartment of the knee. The free fragment was repositioned to its native location and was fixed with three Herbert screws. Also, we found a peripheral avulsion of the posterio-lateral (PL) portion of the lateral meniscus from the capsular attachment, which was repaired with vicryl absorbable suture. An above knee cast was put in 15-degree flexion for three weeks followed by a restoration of knee movements and weight-bearing after ten weeks of surgery. The patient had a full range of motions and no signs of joint laxity after one year of operation.

**Discussion conclusion:**

This study revealed that the surgical repair allowed a quick return to movement for a PLC sheared fracture of the tibial plateau and can be fixed sufficiently to achieve excellent postoperative recovery.

## Highlights

• An avulsion posterio-latero-central tibial plateau fracture was inserted in the anterior compartment of the knee.
• The free fragment was large causing a locking knee but there was no injury for knee ligaments.
• Lateral meniscus was avulsed from the capsular attachment and was repaired with vicryl absorbable suture.
• The fragment was fixed by three Herbert screws with excellent outcome after the surgery.

## 1 Introduction

Tibial plateau fractures are very complex articular fractures with varied fracture configurations and complications [1]. Although tibial plateau fractures represent a rare fracture entity with an approximate incidence of 10.3% and the combination with polytrauma on admission has been estimated at 16–40% [2,3]. The injury mechanism of tibial plateau fractures is largely age-dependent and the majority in the elderly is due to low energy falls. In the younger population, high energy mechanisms predominate and the male gender is more common injured. The injury mechanism can involve motor vehicles, sports, and falls from height [4].

These fractures may constitute a severe injury to the knee joint and complications such as compartment syndrome, post-traumatic arthritis, chronic pain, malunion, and wound problems can develop [4,5]. Soft tissue injuries associated with tibial plateau fractures are frequently seen and up to 45% of patients develop arthritic changes after intraarticular fractures or fractures around the knee joint [6].

Here, we encountered an unusual case of avulsion fracture in the PLC region of the tibial plateau with displacing fragment into the anterior compartment of the knee without any ligamentous injury and the fixation outcome was excellent with Herbert screws. This case report examines one such presentation in line with the SCARE guidelines [7].

## 2 Presentation of case

A 23- year-old male was admitted to our emergency department after injuring his right knee after a crush accident with considerable pain in the injured knee. The patient did not present any history of other diseases. The physical examination revealed that the right knee was slightly swollen, locking in a flexion position of 30°, and the patient was unable to extend his right knee due to severe pain. There was no skin bruise, wounds, or open fractures. A detailed ligamentous exam in the emergency department was deferred due to the acute injury and the patient's significant pain. No other concomitant injuries were detected and vital signs were normal.

**Fig. 1:**
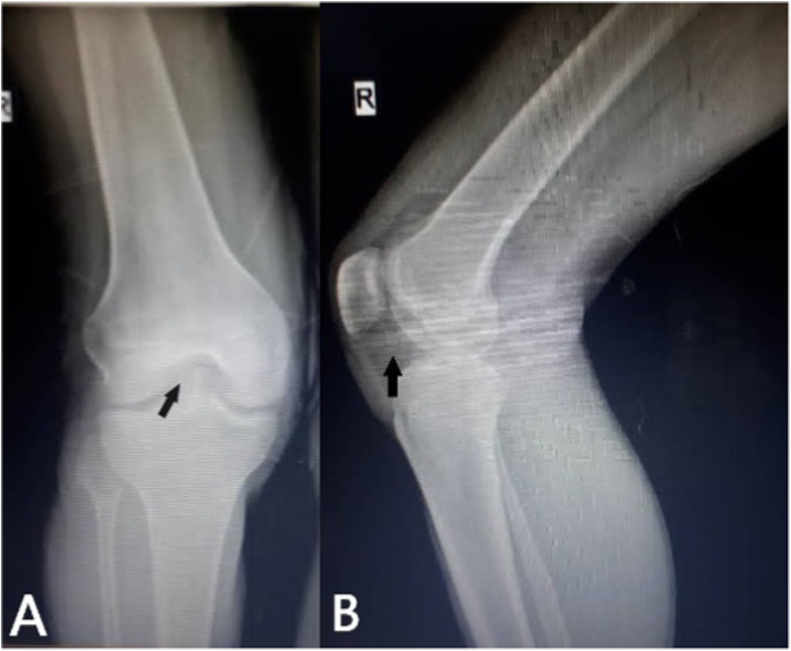
**Anteroposterior (A) and lateral (B) X-ray**s; reveal a shadow into the anterior compartment of the right knee joint (black arrows).

**Fig. 2:**
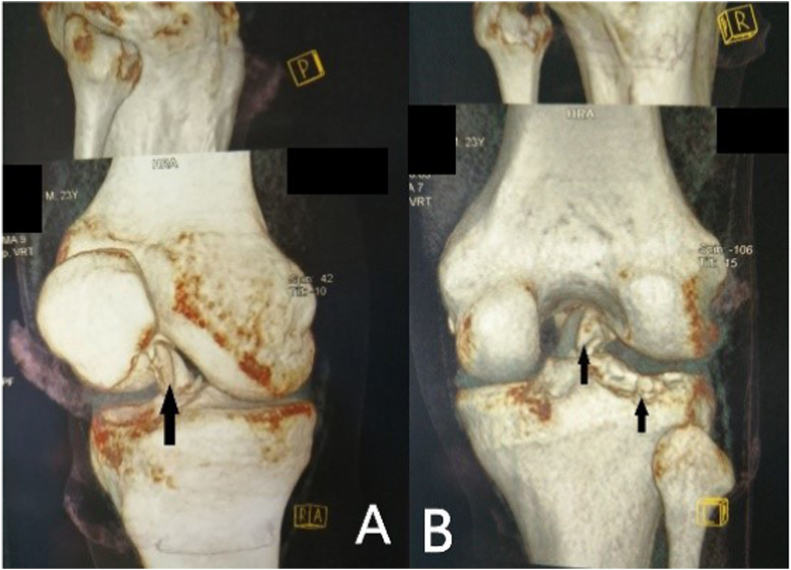
**CT with a reconstruction of the right knee**; anterior (A) and posterior (B) views reveal irregularities in the posterio-latero-central segment of the tibial plateau accompanied by an avulsion fracture displaces and inserts into the anterior compartment of the knee (black arrows).

Anteroposterior (AP) and lateral X-rays (Fig. 1) showed a shadow in the anterior compartment of the right knee joint. CT with a reconstruction of the right knee (Fig. 2) showed irregularities in the PLC segment of the tibial plateau accompanied by an avulsion fracture displaced and inserted into the anterior compartment of the knee.

**Fig. 3:**
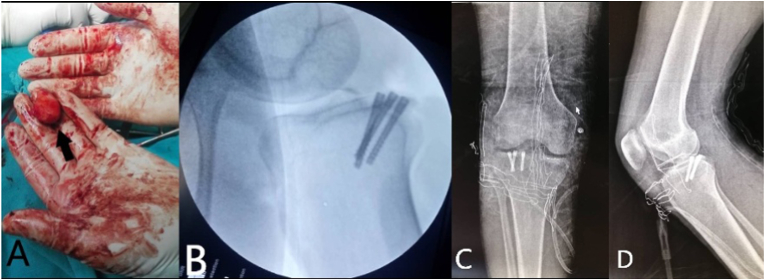
**Intraoperative images; (A);** a large free fragment (3*2 cm) on the surgeon's hand after extraction (black arrow), **(B);** the free fragment fixation with Herbert screws during the surgery. **Immediately postoperative images**; Anteroposterior (A) and lateral (B) X-rays show the fragment repositioning and fixed with three Herbert screws**.**

In the operation room and under epidural anesthesia, the surgery was performed by a fifth-year resident surgeon under the supervisor's view. Prophylactic cefazolin (2 g) was given before the surgery. We did a posterior s-shape approach of the right knee and then opened the capsule. The fragment was extracted by an anterior incision. The free fragment was large (3*2 cm) and was repositioned to its native location and was fixed with three Herbert screws (Fig. 3). During surgery, we found a peripheral avulsion of the posterio-lateral (PL) portion of the lateral meniscus from the capsular attachment, which was repaired with vicryl absorbable suture. The anterior cruciate ligament and posterior cruciate ligament remained intact. The condyle of the femoral articular surface was integrated and smooth. Thereafter, the drainage was inserted and an above-knee cast was put in 15-degree flexion for three weeks.

**Fig. 4:**
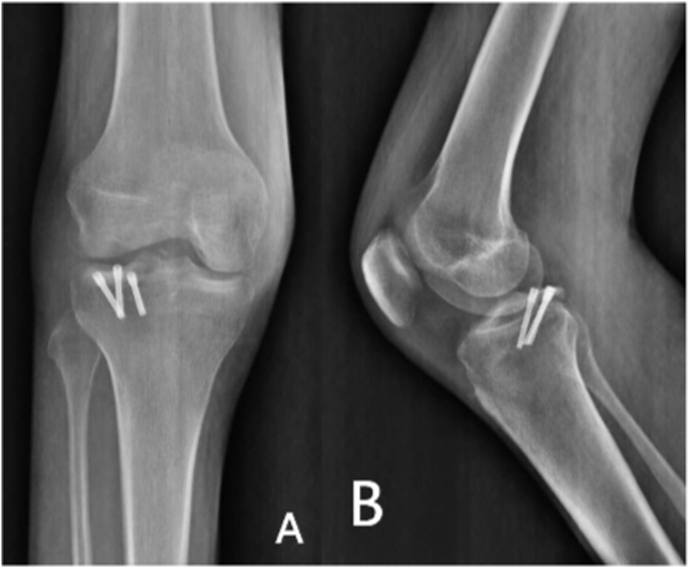
**Anteroposterior (A) and lateral (B) X-rays;** Three weeks after the surgery after removing the cast**.**

After the surgery, the patient was transferred to the Orthopedic ward and discharged after two days with a prescription of apixaban (2.5 mg twice a day) for six weeks. Three weeks later, the cast was removed (Fig. 4), and subsequently, the patient underwent intensive physiotherapy accompanied by progressive weight-bearing. The patient restored knee movements and weight-bearing after ten weeks of surgery.

**Fig. 5:**
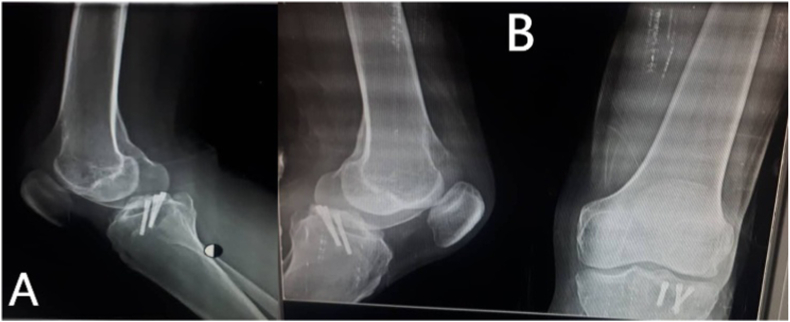
**Anteroposterior (A) and lateral (B) X-rays;** reveal a healed fracture line after two years of the surgery.

**Fig. 6:**
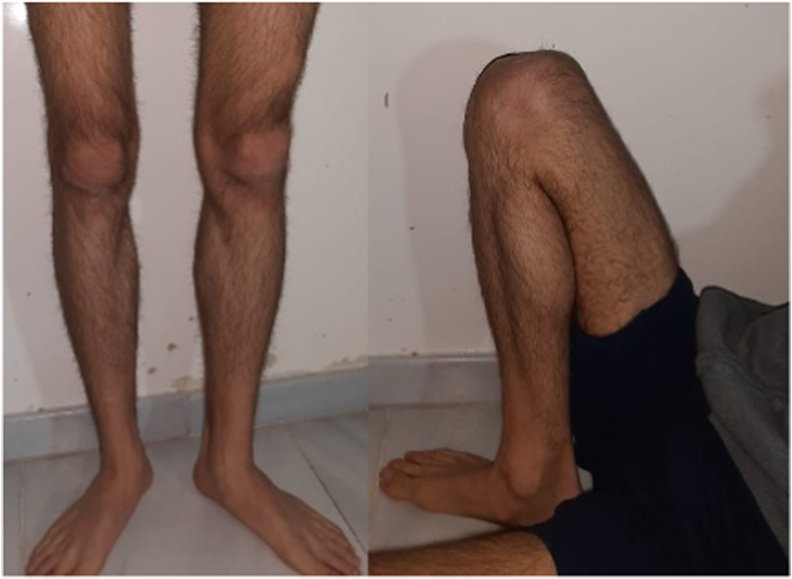
**Clinical images**; showing full extension and flexion of the knee at two years follow up.

The patient's final evaluation was two years after the operation in the orthopedic trauma clinic (Fig. 5). On physical exam, he had a full range of motions and no signs of joint laxity with anterior, posterior, valgus, or varus stress (Fig. 6).

## 3 Discussion

Proximal tibia fractures are the leading cause of damage to the stability and flexibility of the knee [8]. Fractures involving the tibial articular surface account for a little over 1% of all long bone fractures. Several previous classifications were reported for tibial plateau fractures such as Schatzker, Hohl-Moore, Luo, and Orthopedic Trauma Association classifications (OTA/AO) [4]. This case presents a fracture of the segment adjacent to the PL tibial plateau which cannot be easily categorized into the previous classifications.

A more recent cohort study of 246 patient characteristics according to the “Ten segment classification” of the intra-articular tibial plateau fractures based on the OTA/AO classification [5]. In this study, the most frequently affected segment was the PLC (85.9%) in patients with OTA/AO type-C tibial plateau fracture [5]. Also, another study has recently shown that especially the PLC segment is hard to visualize, and satisfactorily reduction under fluoroscopy only was not successful in 89% of the cases involving this segment [9].

Based on the later classification, our patient would be classified as a type-C PLC fracture of the tibial plateau, that required a CT with a reconstruction of the **right** knee to show an avulsion fracture involving this segment, moreover, the free fragment was displaced into the anterior **compartment** of the knee.

Soft tissue injuries occur commonly in tibial plateau fractures. Overall soft tissue injury incidence has been estimated between 73 and 99% from MRI studies [10,11]. Collateral or cruciate injuries were sustained in 77% of patients and lateral and medial meniscus injuries were seen in 91% and 44%, respectively. Overall ligamentous injury incidence has been estimated between 40 and 77% by MRI studies [10,11].

In the current patient, however, the free fragment was large causing a locking knee in a flexion position of 30°, there was no injury of any knee ligaments. Furthermore, a minimal avulsion was observed of the peripheral PL segment of the lateral meniscus from the capsular attachment, which was repaired with vicryl absorbable suture.

To the best of our knowledge, rare variations of the PL tibial plateau fracture with free fragments were reported. The first case described a shear-type of the PL tibial plateau fracture with a fragment inserted into the intercondylar fossa that was fixed with two hollow screws in the addition of Kirschner wires [8]. The second case described two displaced articular fragments detached from the PL corner lying freely in the knee joint where MRI also revealed avulsion of the lateral meniscus from the capsular attachment into the knee joint. These two large fragments were repositioned with Kirschner wires and were fixed using Herbert screws. Also, the avulsed lateral meniscus was sutured to its attachment with fiber wire sutures at multiple places [1].

In our patient, the free fragment was displaced into the anterior **compartment** of the knee which was extracted by the anterior incision. The free fragment was repositioned to its native location and was fixed with three Herbert screws with an excellent outcome where knee movements and weight-bearing were restored after ten weeks of surgery.

## 4 Conclusion

The PLC tibial plateau fractures are severe injuries. We reported on a case in which a large fracture fragment was inserted into the anterior compartment of the right knee with no injury of any knee ligaments and a minimal avulsion of the peripheral PL segment of the lateral meniscus from the capsular attachment. This fragment was repositioned and fixed with three Herbert screws and the lateral meniscus was sutured to its attachment with an absorbable suture. The surgical repair allowed a quick return to mobility and rehabilitation for the patient, which was observed by restored knee movements and weight-bearing after ten weeks of surgery.

## Ethical approval

Written informed consent was obtained from the patient for publication of this case report and accompanying images, in line with local ethical approval requirements and in accordance with the helsinki declaration.

## Sources of funding

This research did not receive any specific grant from funding agencies in the public, commercial, or not-for-profit sectors.

## Author contributions

Abdullah Alsultan writes the manuscript, literature search, treat and follow up the patient and submitted the article.

Mohamad Al Masri writes the manuscript, literature search, treat and follow up the patient.

Mohammad Weam Bolad writes and correct the manuscript, treat and follow up the patient.

Muhammad Al Atrash; manuscript correction, literature search and supervisor of the case.

Mohammad Alsultan writes and correct the manuscript and search the literature.

## Registration of research studies

1. Name of the registry: N\A.

2. Unique Identifying number or registration ID: N\A.

3. Hyperlink to your specific registration (must be publicly accessible and will be checked): N\A.

## Guarantor

The corresponding author is the guarantor of this manuscript.

## Provenance and peer review

Not commissioned, externally peer-reviewed.

## Consent

Written informed consent was obtained from the patient for publication of this case report and accompanying images. A copy of the written consent is available for review by the Editor-in-Chief of this journal on request.

## Declaration of competing interest

The author declares that they have no conflicts of interest regarding this study. The author declares that it has not been published elsewhere and that it has not been submitted simultaneously for publication elsewhere.
